# 

**Published:** 1868-01-01

**Authors:** 


### CHICAGO MEDICAL JOURNAL.

**Edited by J. ADAMS ALLEN, M.D., LL.D.,**

Professor of Principles and Practice of Medicine and Clinical Medicine in Rush Medical College.

All letters for the JOURNAL should be addressed to Dr. J. ADAMS ALLEN, P. O. Box 1948, Chicago, Ill.

TERMS — $3 Per Annum, in Advance.

$4 if not paid before 1st of July, in each year. Single copies, 25 cents.

Rush Medical College — Spring Course of Instruction. The spring course of instruction in this institution will commence on Monday, the 2nd day of March ensuing, and extend through three months. There will be daily recitations and lectures, together with unusual advantages for clinical instruction at the various hospitals of the city and the college dispensary. Details will be given in the next number of the Journal. Full information can be obtained by addressing the Secretary of the college. See advertisement. Prof. Blaney will open his school of instruction in analytical and applied chemistry at the same time, in the laboratory of the college.

